# Effect of BaTiO_3_ on the Properties of PVC-Based Composite Thick Films

**DOI:** 10.3390/ma14185430

**Published:** 2021-09-19

**Authors:** Sarir Uddin, Naheed Akhtar, Sumbal Bibi, Abid Zaman, Asad Ali, Khaled Althubeiti, Hussein Alrobei, Muhammad Mushtaq

**Affiliations:** 1 Department of Physics, Government College Hayatabad, Peshawar 25000, Pakistan; 2 Institute of Chemical Sciences, University of Peshawar, Peshawar 25000, Pakistan; naheedakhter853@gmail.com (N.A.); syedasumbal333@gmail.com (S.B.); 3 Department of Physics, Riphah International University, Islamabad 44000, Pakistan; kasadiiui@gmail.com; 4 Department of Physics, Government Post Graduate College, Nowshera 24100, Pakistan; 5 Department of Chemistry, College of Science, Taif University, P.O. Box 11099, Taif 21944, Saudi Arabia; 6 Department of Mechanical Engineering, College of Engineering, Prince Sattam Bin Abdulaziz University, Alkharj 11942, Saudi Arabia; h.alrobei@psau.edu.sa; 7 Faculty of Materials Science, Beijing University of Technology, Beijing 100124, China; mushtaqphy009@yahoo.com

**Keywords:** polymer, perovskites, composites, dielectrics, PVC/BT

## Abstract

Flexible PVC/BT (Polyvinyl chloride/Barium Titanate) composite thick films with (0–30%) volume fractions of BaTiO_3_ were fabricated via the solution casting method. The effects of BaTiO_3_ filler on the phase, microstructure and dielectric properties of composite films were investigated. The XRD results revealed that BT particles are embedded in the PVC matrix with no chemical reaction taking place between the two phases. It was observed that the glass transition temperature of PVC had increased with the addition of BT. The frequency dispersion in the dielectric constant versus temperature curves indicated the relaxor nature of the composites. The dielectric constant (ε_r_) measured at 40 °C, increased from 7.6 for pure PVC to 16.1 for 30% of BaTiO_3_ content in PVC polymer matrix. It is suggested that BaTiO_3_ ceramic powder enhanced the dielectric properties of PVC and may be used as a flexible dielectric material.

## 1. Introduction

Dielectric materials are used extensively as piezoelectric transducers and actuators, as ferroelectric memory and energy storage devices, or as dielectric antenna and filter in wireless telecommunication devices [1,2,3]. The Electronic Industries Alliance (EIA) classifies dielectric capacitors into different categories depending on the dielectric constant (ε_r_) of the dielectric medium [4]. The dielectric constant determines the amount of energy that a capacitor can store compared to vacuum [5]. Dielectric capacitors are categorized into three sub-classes depending on their thermal and dielectric properties. Class-1 dielectrics are commonly used in capacitors, exhibiting temperature-stable performance, low acoustic noise and low dielectric loss or high quality factor. These dielectrics usually exhibited intermediate values of dielectric constant (15–500), lower dissipation factor and negligible aging effect [6]. Class-1 dielectric mainly includes para-electrics such as MgTiO_3_, MgNb_2_O_6_, BaTi_4_O_9_ and their substructure ceramic compounds. Apart from useful properties of ceramic dielectrics, these are brittle and cause difficulty in the fabrication of complex shapes or can break during moving components in electromechanical systems. On the other hand, polymers are flexible and can be turned into any required shape. The prime drawbacks of polymers are their low dielectric properties, which limits their applications in electronic industries [7,8]. One of the solutions may be the fabrication of ceramic/polymer composites. Ceramic/polymer composites are investigated broadly due to easier processing, good chemical stability and useful mechanical and dielectric properties [9,10,11,12,13]. Ceramic/polymer composites combine the better dielectric properties of the ceramic powders (filler) and the mechanical flexibility, chemical inertness and shape-forming possibility of polymer (matrix).

Among polymers, polyvinyl chloride (PVC) is an amorphous piezoelectric thermoplastic polymer which is formed from the C_2_H_3_Cl (vinyl chloride) monomer to a long chain [(C_2_H_3_Cl)n] polymer [14,15]. The piezoelectric coefficients d_31_ of PVC has been reported in the range of 0.5 to 1.3 pC/N with a glass transition temperature of 80 °C [16,17]. PVC possess low dielectric constant (ε_r_ = 4) and can be modified by adding various piezoelectric ceramic powders [18,19,20,21,22]. Funt has put forward the microwave dielectric properties of PVC under radio frequencies [23]. Amrhein and Mueller have studied the microwave dielectric measurements of PVC and its derivatives [24]. Perovskite-structured BaTiO_3_ ceramic possesses higher dielectric constant (~4500) and stable tetragonal structures with the space group (14/m) at room temperature with a saturation polarization of 16 µC/cm^2^ [25,26]. Olszowy investigated the microwave dielectric properties of PVC/BT composites fabricated through hot pressing method [20]. Many studies reported the effect of BT ceramic on the dielectric properties of polymer [27]. Recently, Berrag et al. used Cole-Cole’s model to validate the experimental microwave dielectric data of PVC/BT composites [22].

In this paper, we have investigated the structural, morphological and dielectric properties of PVC/BT composite thick films with different BT contents (10%, 20% and 30%) as filler in the PVC matrix.

## 2. Materials and Synthesis

**Figure 1:**
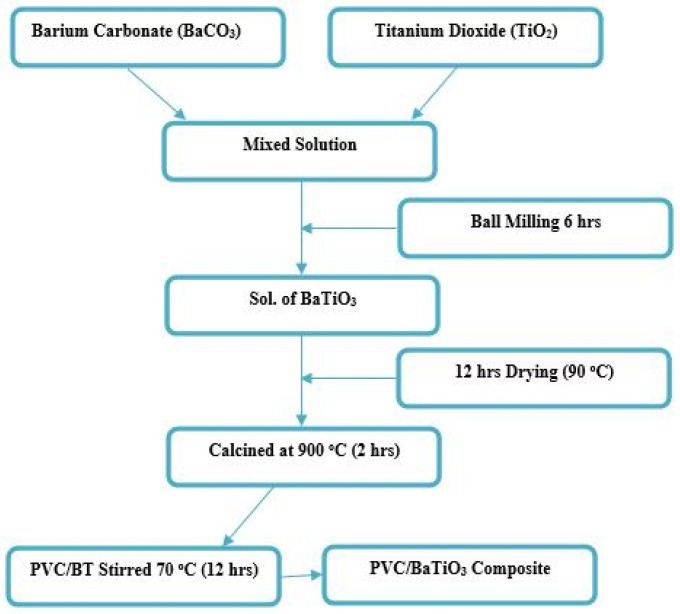
A schematic representation for the preparation of BT, PVC and PVC/BT composites.

BaTiO_3_ ceramic powder was synthesized using research-grade BaCO_3_ and TiO_2_ via the solid-state route (Figure 1). These reactants were weighed in stoichiometric ratios and mixed/milled for 6 h in polyethylene bottle. For grinding media, we used y-toughened zirconium balls and to make free flowing slurry, ethanol was added as a lubricant. The slurry was then dried in an oven for 12 h at 90 °C. Moreover, the dried reactant powders were calcined at 900 °C for 2 h at a heating/cooling rate of 5°C/min in air. In order to obtain fine powder, the calcined BT powder was ground with pestle and mortar.

PVC/BaTiO_3_ composite films were synthesized via the solution casting method. Different volume fractions of PVC were dispersed in Di-Methyl formamide (DMF) using an ultrasonicator for 30 min. BaTiO_3_ powder was added in different portions and magnetic stirred at 70 °C for 12 h to obtain uniform PVC/BaTiO_3_ suspension. The PVC/BaTiO_3_ suspension was then casted onto a flat aluminum sheet (3 cm × 3 cm) which is then kept in an oven at 70 °C for 30 min to obtain dried composite thick films. In order to evaporate the DMF, the composite films were further heated at 110 °C in a vacuum furnace for one hour. A portion of the PVC/BT composites films were then peeled off from the aluminum substrate for further characterization.

## 3. Characterization

The phase analysis was carried out via x-ray diffractometer (XRD) (JDX-3532, JEOL, Tokyo, Japan) with Cu (Kα) radiation with wavelength (λ = 1.5418 Å). The Fourier transformed infrared radiation (FTIR) spectra were obtained using an FTIR spectrometer. The microstructural study of the PVC/BT composite thick films were carried out via the secondary electron field effect, scanning electron microscope (SEM) (JEOL 6400 SEM, Tokyo, Japan). The dielectric properties were measured by LCR meter (HP 4192A) using a coated silver layer on top surfaces of composite films as electrodes.

The dielectric constant ($\varepsilon^{\prime }$) and dielectric loss (tan δ) was calculated by using the formula [26]:

$$\varepsilon^{\prime }=\frac{d}{A\varepsilon_{o}}C$$

$$tan\;\delta =\frac{\varepsilon^{\prime \prime }}{\varepsilon^{\prime }}$$

where $\varepsilon^{\prime \prime }$, d, A, C and $\varepsilon_{o}$ are the imaginary part of the dielectric constant, the sample thickness, area of sample, the capacitance of sample and the permittivity of free space (8.85 × 10^−12^ F/m), respectively.

## 4. Results and Discussion

### 4.1. Phase and Microstructural Analyses

**Figure 2:**
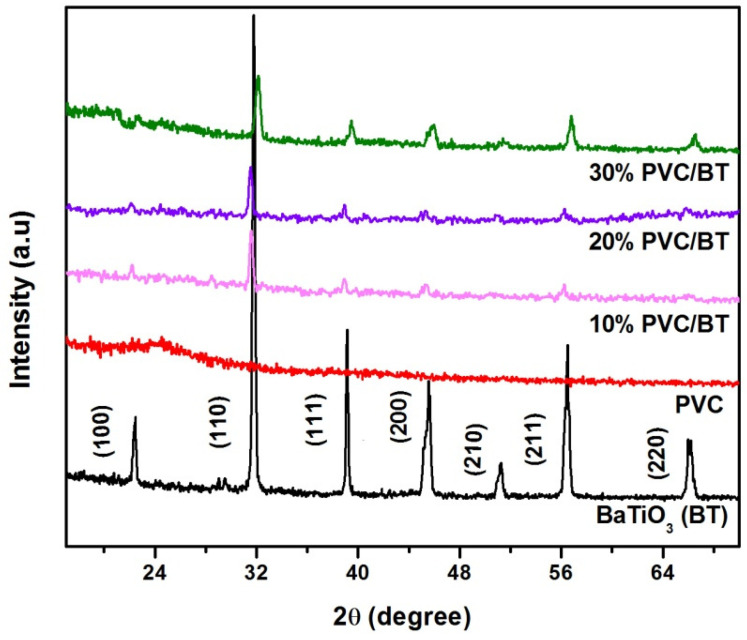
XRD patterns of BT, PVC and PVC/BT composites with various BT content in PVC matrix.

The room temperature XRD patterns of BaTiO_3_, PVC and PVC/BT composites with various BaTiO_3_ contents as filler are shown in Figure 2. The XRD pattern of BT revealed the formation of a tetragonal (14/m) perovskite structure with no impurity phase [27]. The XRD pattern for pure PVC indicated the amorphous nature consistent with previous studies [18,20]. The XRD patterns of PVC/BT composite films indicated the stability of BT in the PVC matrix. These results suggest that the single-phase crystalline powders of BT are embedded in PVC matrix. The diffraction peaks of BT become stronger gradually with the increase in BT content in the PVC/BT composites. XRD results suggest that BaTiO_3_ ceramic powder maintains its crystalline nature in the composite thick films and are completely coated by PVC matrix.

**Figure 3:**
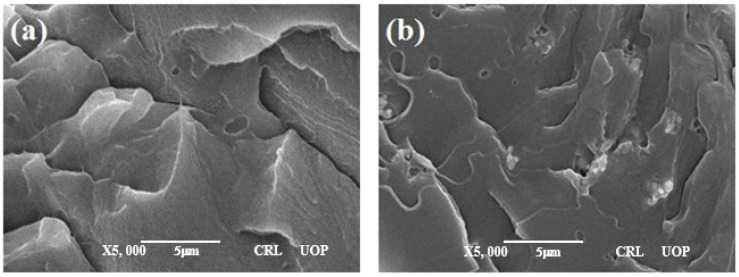

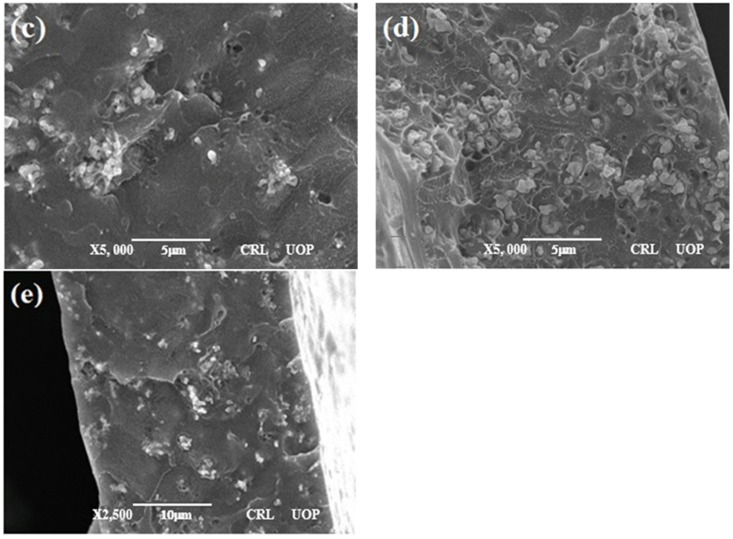
SEM images of PVC/BT composite thick films. (**a**) 0%, (**b**) 10%, (**c**) 20%, (**d**) 30%, (**e**) cross sectional view showing the thickness of films.

The secondary electron scanning electron microscope (SEM) images of PVC/BT composite thick films are shown in Figure 3. SEM images indicated that the pure PVC acted as a host matrix material and BaTiO_3_ particles are almost evenly distributed with minimum agglomeration. Figure 3a for pure PVC revealed smooth surface morphology consistent with previous studies [18,19,20,21]. The distribution of fillers in 30% of BT content is more obvious than that of 10% and 20% of BT content in the host PVC matrix. The fabricated PVC/BaTiO_3_ composite films are translucent and homogeneous, which proves the formation process for obtaining flexible composite thick films. Figure 3e indicates the thickness of the films to be around 25 µm on average.

### 4.2. FTIR Analysis

**Figure 4:**
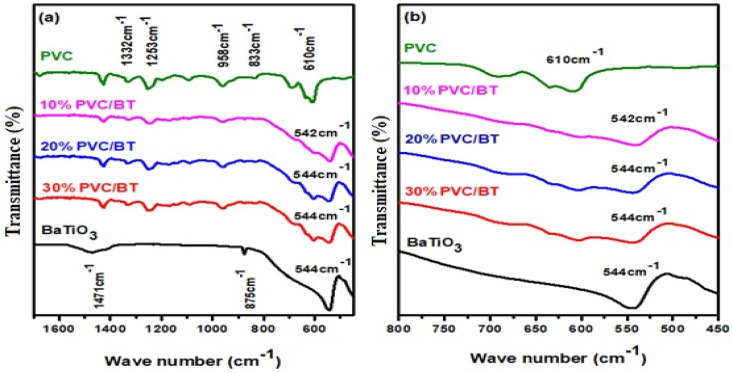
(**a**) FTIR absorption spectra of BT, PVC and PVC/BT composites with various BT content in PVC matrix; (**b**) shows that the relative intensities of peaks are decreased with the decrease in PVC content.

The FTIR absorption spectra of BT, PVC and PVC/BT composite thick films with various BT content as fillers are shown in Figure 4a. For pure PVC, the characteristic absorption peaks of CH_2_ deformation mode were observed at 1332 cm^−1^, CH rocking mode at 1253 cm^−1^, trans CH-wagging mode at 958 cm^−1^, C-Cl stretching mode at 833 cm^−1^ and C is CH-wagging mode at 610 cm^−1^, consistent with previous reports [18,21]. For pure BT, a broad peak is observed at 544 cm^−1^ due to the O-Ti-O vibration [28].

Figure 4b shows that the relative intensities of peaks are decreased with the decrease in PVC content in the composite films. The peaks at 1332 cm^−1^, 1253 cm^−1^, 959 cm^−1^ and 833 cm^−1^ did not shift in composite films, which indicates that there is no chemical reaction taking place between the two phases [21]. The peak at 610 cm^−1^ has changed from a sharp peak at a lower wave number to a broader peak at 544 cm^−1^ with increasing BaTiO_3_ content. This change in peak might be due to the overlapping with the strong peak at 544 cm^−1^ originating from BaTiO_3_ or due to the overlapping of two peaks at closer wave numbers.

### 4.3. Dielectric Properties

**Figure 5:**
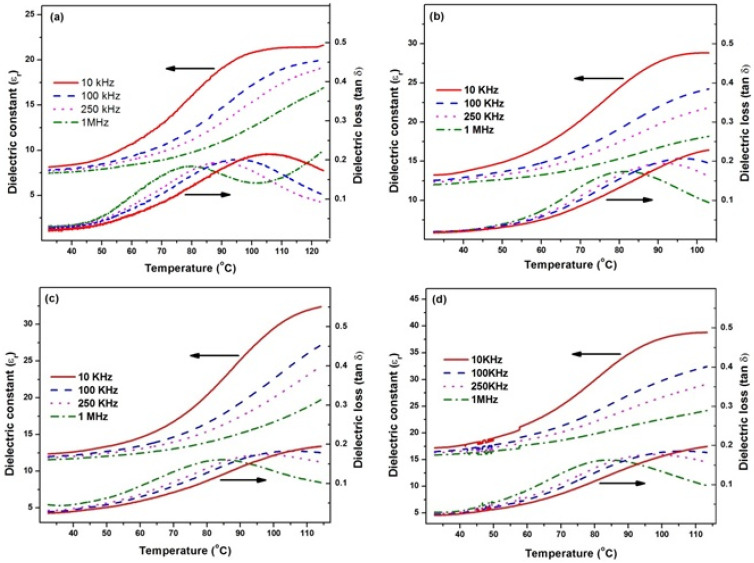
Temperature and frequency dependence of ε_r_ and tan δ for various BT content in PVC matrix. (**a**) 0%, (**b**) 10%, (**c**) 20%, (**d**) 30%.

The plots of dielectric constant (ε_r_) and dielectric loss (tan δ) versus temperature of PVC/BT composites at various frequencies are shown in Figure 5. In the ε_r_ and tan δ versus temperature curves, anomalies were observed at about 80 °C for PVC and at about 100 °C for PVC/BT composites. These anomalies may be attributed to two factors. One factor is the glass transition temperature of PVC, which is 80 °C, and the second factor is the curie point of BaTiO_3_, which is 120 °C [16]. At the glass transition temperature, the polymer transforms from a hard semi-crystalline structure to a soft rubbery form. At the Curie point (T_c_), BaTiO_3_ transforms from the non-centro-symmetric ferroelectric phase to the centro-symmetric paraelectric phase [29]. Around the Curie point, every ferroelectric exhibits higher dielectric constant [30,31]. The ε_r_ was observed to decrease with increasing frequency and increased with increasing temperature. At higher temperatures, the dipoles of molecules orient themselves more easily along the applied electric field and cause an increase in the dielectric constant [32]. The dielectric constant increases with the increase in BT content in the fabricated PVC/BT composites, which may be attributed to the higher ε_r_ values of BT ceramics [33,34]. The dielectric constant (ε_r_) at 40 °C for 1 MHz of the composites increases from 7.6 to 16.1 with increasing BT content. The increase in electronic conduction with increasing temperature leads to an increase in dielectric losses (tan δ) of PVC/BT composites [35].

**Figure 6:**
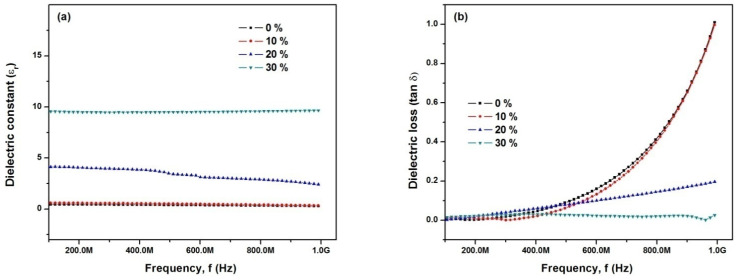
(**a**) Variation in dielectric constant (ε_r_) with frequency (f) (**b**) Variation in dielectric loss (tan δ) with frequency (f) for various BT content.

The frequency dispersion in ε_r_ and tan δ versus temperature curves indicates relaxor behavior of fabricated samples, shown in Figure 5. These curves demonstrated typical relaxor behavior with the magnitude of the dielectric constant decreasing with increasing frequency and the peaks of these curves were shifted to higher temperatures [36,37]. Smolenski [38] proposed that underlying the relaxor behavior was a chemical inhomogeneity on a cation site, giving rise to a diffuse phase transformation (DPT). Randall [39] has found evidence for short-range chemical order on the nano-scale level using transmission electron microscopy (TEM). It is proposed that chemical inhomogeneity at the nano-scale causes the relaxor behavior [40]. The variation of ε_r_ and tan δ with frequency (f) for various BT contents are shown in Figure 6. The orientation polarization decreases with increasing frequency and results in a decrease in ε_r_, which may be attributed to time lagging between flipping dipoles and applied electric field [41].

## 5. Conclusions

PVC/BT composites were synthesized via the solution casting method. The synthesized samples’ structural, vibrational, morphological and dielectric properties of PVC/BT composite thick films were investigated. The phase analysis of PVC/BT composites indicated that the single-phase crystalline powders of BaTiO_3_ are embedded in the PVC polymer matrix, resulting in a two-phase composite material. The microstructural analysis revealed that BT particles are dispersed in the PVC matrix, with no chemical reaction taking place between the fillers and matrix. The spectra of BT, PVC and PVC/BT composite thick films represent a common peak at 544 cm^−1^, 610 cm^−1^ and 542 cm^−1^, which can be estimated as a stretching mode of the C-Cl/CH-wagging group, presumably adsorbed at surface. The temperature-dependent dielectric properties of PVC/BT composite films indicated frequency dispersion and improvement with an increase in BT ceramic filler in PVC matrix.

## Data Availability

Data is contained within the article.
